# Who Benefits from Barefooting? The Key Role of Baseline Wellbeing in Psychophysical Restoration

**DOI:** 10.3390/ijerph22121779

**Published:** 2025-11-25

**Authors:** Aurelia De Lorenzo, Samuele Berteotti, Fabrizia Giannotta, Emanuela Rabaglietti

**Affiliations:** Department of Psychology, University of Turin, 10127 Turin, Italy; aurelia.delorenzo@unito.it (A.D.L.); samuele.berteotti@unito.it (S.B.); fabrizia.giannotta@unito.it (F.G.)

**Keywords:** barefooting, nature-based activities, mental wellbeing, perceived stress, psychophysical recovery

## Abstract

Nature-based activities have been linked to psychophysical restoration, but the role of individual baseline conditions in predicting recovery remains unclear. This study examined whether baseline stress levels and mental wellbeing influence psychophysical recovery after an immersive barefooting experience, and whether sociodemographic factors (sex and education) and access to green spaces moderate these effects. A convenience sample of 249 adults (58% female, mean age 45 years) voluntarily participated in a structured barefooting trail in two Italian parks and, after the activity, completed post-experience self-report questionnaires using validated scales (Perceived Stress Scale, Restorative Outcome Scale, and Warwick–Edinburgh Mental Wellbeing Scale). Multivariate logistic regression analyses showed that higher baseline mental wellbeing significantly predicted greater psychophysical recovery, while short-term perceived stress did not. None of the tested interactions with sex, education level, or access to green space were significant. These results suggest that mental wellbeing, rather than recent stress, may be a key factor in maximizing restorative experiences in immersive nature-based activities, and that this effect appears consistent across sociodemographic and environmental contexts. While preliminary, these findings highlight the potential of barefoot walking and similar multisensory activities as low-cost strategies to maintain and enhance psychological wellbeing.

## 1. Introduction

People often seek recreational activities in natural settings to manage daily stress and promote psychological wellbeing. As urban living and the pace of modern life contribute to higher levels of perceived stress and declining mental health, green spaces—such as parks, woodlands, and nature reserves—provide accessible venues for restorative experiences [1]. During the COVID-19 lockdowns, outdoor recreation increased dramatically—by as much as 291% in Oslo [2] and fourfold in parts of China [3]—suggesting that people turn to nature especially when under heightened strain. A robust body of empirical research has documented that contact with natural environments yields benefits across emotional, cognitive, and physiological domains. For example, a literature review found evidence for associations between exposure to nature and improved cognitive function, brain activity, blood pressure, mental health, physical activity, and sleep [4]. Spending at least 120 min per week in nature correlates with better overall health and wellbeing [1], while park and forest visits elicit measurable decreases in cortisol, increases in heart-rate variability, and subjective reports of relaxation [5,6,7]. These findings underscore the contribution of nature-based recreational activities to psychological and physical wellbeing.

Two leading theories shape our understanding of how nature produces these restorative effects. Kaplan’s Attention Restoration Theory (ART) proposes that prolonged use of directed attention—required for tasks involving cognitive control and sustained focus—can lead to attentional fatigue. According to ART, natural environments promote recovery by offering softly fascinating stimuli that effortlessly engage attention without requiring cognitive effort, allowing the directed attention system to rest and recover [8]. Unlike urban settings, which often demand constant vigilance and cognitive filtering, natural stimuli provide a restorative experience that supports cognitive functioning and mental wellbeing. Complementing this cognitive perspective, Ulrich’s Stress Reduction Theory (SRT) focuses on the affective and physiological benefits of nature exposure. SRT suggests that natural environments can elicit immediate positive emotional responses and activate parasympathetic processes that reduce stress and physiological arousal [9]. While both theories emphasize the restorative effects of nature, ART centers on cognitive recovery through attentional mechanisms, whereas SRT highlights rapid affective and physiological stress reduction.

Despite extensive evidence for the benefits of passive or low-intensity nature exposure (e.g., viewing landscapes, walking on paved trails), less is known about more immersive, sensorimotor forms of engagement. Embodied-cognition research indicates that multisensory stimulation amplifies connectedness to nature, which predicts life satisfaction and deepens restorative experiences [10,11]. One example of an activity that allows multi-sensorial experience is barefoot walking. The tactile engagement with natural substrates involved in this activity appears to activate sensory pathways that are less stimulated during shod walking or exposure to virtual nature. For instance, Koga and Iwasaki [12] found that barefoot contact with grass surfaces significantly modulates cerebral blood flow in the prefrontal cortex—an area linked to executive functions such as planning, emotional regulation, and social cognition. These shifts suggest a downregulation of cognitive effort and a transition toward a more relaxed, parasympathetic-dominant state. Similarly, Ikei et al. [13] reported that direct foot contact with natural materials induces measurable changes in autonomic nervous system activity, promoting physiological states associated with relaxation and stress recovery. These findings align with the effects observed by Ulrich during exposure to natural visual stimulation and those suggested by ART regarding exposure to non-specific natural stimulation [9].

Despite the wealth of evidence on the positive effects of intense nature exposure activities, less is known about how individuals’ initial mental condition affects effectiveness. For instance, Kaplan [8] reported that participants with high mental fatigue exhibited greater attention recovery after a nature walk than those with low-fatigue, and Ulrich et al. [9] observed steeper declines in physiological stress markers among individuals who entered studies with elevated blood pressure and cortisol after a 10 min exposure to a natural scenery.

Similarly, general mental health—often measured by self-report measures of wellbeing and the absence of common mental disorder (CMD) symptoms—can influence individuals’ ability to engage with and benefit from environmental interventions. People with lower mental health scores may experience a paradox: although they face greater barriers to accessing and enjoying natural spaces, such as lower intrinsic motivation to visit nature, reduced visit-related happiness, and increased visit-related anxiety [14], when they do engage they often report greater affective improvements and deeper cognitive restoration [15,16]. This pattern reflects what Berman and colleagues [15] called a “restoration gap,” in which distressed individuals gain disproportionately larger benefits, partially closing the gap between affected and unaffected groups in post-experience functioning. Additionally, studies have found that improvements in mood and memory occur through independent mechanisms [15], suggesting that nature may influence wellbeing and cognitive functioning through potentially separate pathways, with different magnitudes, in parallel but not necessarily interconnected ways.

Psychological states, which can influence how people approach and benefit from barefoot walking, also vary according to personal characteristics such as gender and socioeconomic status. For example, some evidence suggests that gender differences exist in both stress perception and stress reactivity, with women often reporting higher average levels [17,18]. Additionally, research indicates that individuals with lower socioeconomic status (SES) often experience worse mental health outcomes than those with higher SES. Specifically, people who are privileged tend to have better mental health, while those facing deprivation are at greater risk for serious mental health problems, forming a well-documented gradient in health disparities [19].

In addition to psychological states and personal characteristics, certain situational and environmental factors also play a role, such as access to natural green spaces, which influences habitual engagement. People who live near forests or parks tend to visit them more often and may therefore experience smaller marginal gains compared to those who live farther away, who may find a single, immersive walk more effective [20,21]. Including these moderators allowed us to test whether unstable traits such as baseline stress and mental wellbeing predict psychophysical restoration from barefoot walking, while accounting for stable traits such as sex, SES, and access to nearby green spaces. The significant increase in outdoor activities worldwide that began during the COVID-19 lockdowns boosted the scientific community’s efforts to evaluate the positive effects of interaction with nature. While many studies have already focused on these positive effects, much remains to be investigated regarding the interaction between stable personal characteristics and health conditions when seeking the highest possible restoration through outdoor activity. In this study, we focus on people who spontaneously seek restoration through a barefoot walking experience, an immersive activity in a high-quality natural context.

In summary, using a sample of individuals who participated in a barefoot walking activity in nature to achieve psychophysical restoration, this study aims to:

1. Investigate the extent to which baseline stress level predicts the probability of achieving elevated levels of psychophysiological restoration.
2. Investigate the extent to which baseline mental wellbeing predicts the probability of achieving elevated levels of psychophysiological restoration.
3. Explore the moderating role of SES level, biological sex, and accessibility to natural green spaces, on the relationship between baseline stress level and probability of achieving elevated levels of psychophysiological restoration.
4. Explore the moderating role of SES level, biological sex, and accessibility to natural green spaces on the relationship between baseline mental wellbeing level and the probability of achieving elevated levels of psychophysiological restoration.

## 2. Materials and Methods

### 2.1. Procedure

This study was conducted with a convenience sample of adults who participated in the project by first completing the barefoot trail and then filling out a self-report questionnaire. Participants voluntarily visited two parks in the Piedmont region that promoted the sensory trail “La foresta in punta di piedi” (The forest on tiptoes), funded by the European Regional Development Fund as part of the ALCOTRA NAT.SENS 2014–2020 project, financed by the European Union. This cross-sectional study collected data through a self-report questionnaire, available digitally via the Limesurvey platform and in paper form for those without a personal device. Trained university staff administered the questionnaires at the association’s sites. The university, through its media contacts and channels, invited all age groups to participate in the project, from students to senior citizens involved in parallel projects. Although the sample was self-selected on a voluntary basis, broad participation from different age groups and types of participants was achieved. All participants accessed the barefoot trails by paying a registration fee and voluntarily chose to participate in the study by completing the questionnaire. The questionnaire was administered in spring and summer 2024.

The study was conducted in accordance with the Code of Ethics of the World Medical Association (Declaration of Helsinki), which outlines ethical principles for research involving human subjects [22]. After signing the informed consent form, participants voluntarily completed the questionnaire in a maximum of 20 min. All data were collected anonymously in accordance with research ethics regulations. The protection of personal data is guaranteed by specific legislation (Legislative Decree 101/18, 679/2016, General Data Protection Regulation) and by the researchers responsible for the project. This study was also approved by the Bioethics Committee of the University of Turin (Prot. no. 0432955 of 21 July 2023).

### 2.2. Barefooting Activity Description

To describe the barefooting activity proposed in this study, the CERT (Consensus on Exercise Reporting Template) by Slade and colleagues [23] was used as a reference. The main items of the template are listed below:

WHAT: The intervention involved a barefoot walking activity on natural ground, requiring no specific equipment. Participants were organized into small groups (maximum of 10 participants, average of 6) and accompanied by a trained and certified Natural Guide from the sensory trail “La foresta in punta di piedi” (The forest on tiptoes). The guide ensured participants’ motivation, engagement, and the overall quality of contact with nature throughout the experience. Each activity along the trail was introduced and contextualized by the guide, who explained its purpose and meaning. As the experience was both guided and voluntary, measurement or reporting of adherence was considered unnecessary.

WHO: The subjects who participated in the pathway are described in the “participants” section (see Section 2.3).

WHERE: The barefooting activity was conducted in two parks of the protected areas in the royal parks of the Piedmont region, Italy, where the sensory trail “La foresta in punta di piedi” (The forest on tiptoes) was held, funded by the ALCOTRA NAT.SENS Project 2014–2020 European Regional Development Fund, financed by the European Union.

WHEN/HOW LONG: The experiment was conducted in warm or mild weather, between May and September. Each guided session lasted between 90 and 120 min.

TAILORING AND STANDARDIZATION: All participants experienced the same 600 m sensory trail under comparable environmental conditions. Each group walked the entire trail barefoot, accompanied by an expert nature guide. Ten dedicated stations were evenly distributed along the trail, each designed to focus participants’ attention on different sensory aspects of interacting with the natural environment. The ten activities included: walking on stones, walking on ground of different temperatures, walking on hay, walking on a floor of horizontal logs, walking in water, walking in mud, walking on vertical logs of different heights, walking on a slackline, walking with eyes closed, and collecting sticks with the feet. Throughout the experience, the guide continuously shared knowledge and insights about the natural and historical features of the trail and surrounding park, helping participants stay engaged and preventing their attention from drifting back to everyday concerns before the activity ended.

PROCEDURE: All respondents to the questionnaire participated in a single session of barefooting. Before the activity began, all participants were informed about the experimental study and the subsequent survey and signed consent forms for privacy and participation. Immediately after completing the course, the questionnaire was administered digitally (to be completed on a smartphone) or in paper form for those who requested it. The questionnaire was administered within the park, in a dedicated area, and was supervised by university researchers specially trained for this purpose.

MONITORING AND DATA QUALITY: Both the Natural Guides and the researchers monitored the entire process to identify potential issues. No systemic problems were reported. A small number of participants discontinued the activity for personal reasons and were excluded from the analysis. However, those who ended participation early due to discomfort with the barefoot experience were retained in the final dataset (less than 20 in total). All remaining participants completed the activity as planned.

### 2.3. Participants

The study included 249 adults, 58% of whom were biologically female (*n* = 144), with a mean age of 45 years (age range = 22–84; SD = 18.38). All participants were born in Italy, except for one individual born in Romania. Fifty-nine percent held a higher education degree (Bachelor’s or Master’s). Forty-two percent reported living in an environment with access to green spaces such as parks, nature reserves, forests, the sea, rivers, or lakes.

### 2.4. Measures

The measurements of stress and mental wellbeing refer to a specific period, namely the last 15 days. In contrast, the measurement of psychophysical recovery, was administered to participants with explicit reference to the fact that each item relates to recovery immediately after the barefoot activity.

#### 2.4.1. Perceived Stress

The original 14-item Perceived Stress Scale (PSS-14) was developed in 1983 by Cohen et al. (1983), but this version was later revised and reduced to a 10-item version [24]. The Italian version of the PSS-10 [25] consists of 10 items and was used to measure the extent to which life was unpredictable, uncontrollable, and overwhelming in recent weeks. It is based on a 5-point Likert scale (0 = “never”, “1 = “almost never”, “2 = “sometimes”, “3 = “quite often”, “4 = “very often”). Example items include ‘How often have you felt that you were unable to control the important things in your life?’ and ‘How often have you been upset because of something that happened unexpectedly?’ After reversing the scores for the four positively formulated items (items 4, 5, 7 and 8), a total PSS-10 score was calculated by summing all the items. Higher scores indicate a higher perception of stress. The psychometric properties of the PSS-10 have been examined in different countries and target groups, supporting the use of the scale as a reliable and valid measure of perceived stress, e.g., [26,27,28,29]. In the present study, Cronbach’s alpha was 0.82.

#### 2.4.2. Psychophysical Recovery

Restorative Outcome Scale (ROS): The Restorative Outcome Scale (ROS) [30] is used to assess restorative emotional and cognitive outcomes in a given environment through six items such as ‘I feel calmer after being here’, ‘I receive new enthusiasm and energy for my daily routines from here.” Each item is rated on a seven-point Likert scale, from 1 (not at all) to 7 (completely), with reliability confirmed by previous research, e.g., [31]. In this study, since an Italian version was not available, we translated the original English version into Italian using a five-stage test adaptation procedure [32]: (1) two independent forward translations; (2) synthesis of the translations; (3) back-translation by two independent translators; (4) review by an expert committee to ensure semantic and conceptual equivalence; (5) pretesting of the pre-final version on a sample from the target population. In the present study, Cronbach’s alpha was 0.92.

#### 2.4.3. Mental Wellbeing

Mental wellbeing was assessed using the Warwick–Edinburgh Mental Wellbeing Scale (WEMWBS) [33]. The Italian version of the WEMWBS [34] is a self-report questionnaire that uses a Likert scale from 1 (not at all) to 5 (always) to measure positive attitude toward life over the past two weeks through 12 items, such as ‘I’ve been feeling optimistic about the future’ and ‘I’ve been dealing with problems well’. This instrument evaluates mental wellbeing as a stable presence of positive states, rather than the absence of negative symptoms or stress. The scores for the 12 items were summed to create a total score, with higher scores indicating a higher level of mental wellbeing. The reliability of the Italian version was assessed by Gremigni and Stewart-Brown [34], with Cronbach’s Alpha between 0.83 and 0.86. In the present study, Cronbach’s alpha was 0.90.

#### 2.4.4. Familiarity with Nature

Familiarity with nature was measured using an index of four items in which respondents indicated how often they visit nature for hikes or trips to parks, nature reserves, mountains, forests, rivers, lakes, or the sea. The items were rated on a Likert scale from 1 to 4, ranging from “never” to “1 or more times a month.” Higher scores indicate greater familiarity with natural environments. In the present study, Cronbach’s alpha was 0.84.

#### 2.4.5. Moderators and Covariates

Participants were asked to indicate their biological sex (0 = female, 1 = male, 2 = no response), age (numerical response), level of education (1 = elementary, middle, or high school diploma; 2 = bachelor’s, master’s degree, or Ph.D.), and access to green spaces (yes = 0, no = 1). In this study, level of education was considered a proxy for socioeconomic status, as suggested by the literature [35].

### 2.5. Statistical Analysis

Statistical analysis was performed using SPSS version 29 [36].

To examine how the initial level of perceived stress and/or mental wellbeing predicted levels of psychophysical recovery, we conducted a series of multivariate logistic regression analyses. The models included demographic factors such as age and familiarity with nature. Assumption checks confirmed that there were no violations of multicollinearity. The dependent variable, psychophysical recovery, was dichotomized based on the mean score (cutoff = 33.72), with scores above the mean categorized as “high recovery” and scores below as “low recovery”.

The initial model included the following predictor variables: age, familiarity with nature, perceived stress (PSS), mental wellbeing (WEMWBS), sex (coded as 1 = female, 2 = male), education level (Low vs. high, as described in Moderators and covariates), and access to green spaces (yes or not, as described in Moderators and covariates). We first tested whether the initial levels of perceived stress and/or mental wellbeing predicted levels of psychophysical recovery. 

Next, six additional models were tested to explore interaction effects. The first three models included an interaction term between stress level and sex, education level, and ease of access to green spaces (1 = stress × sex; 2 = stress × education; 3 = stress × access to green spaces). The last three models included an interaction term between wellbeing and sex, education level, and ease of access to green spaces (4 = wellbeing × sex; 5 = wellbeing × education; 6 = wellbeing × access to green spaces).

## 3. Results

### 3.1. Descriptive Analyses

The descriptive data are presented in Table 1.

There were no statistically significant differences in mental wellbeing scores by biological sex (*t*(247) = 0.99; *p* = 0.32), education level (*t*(247) = 0.83; *p* = 0.40) or ease of access to green spaces (*t*(247) = 1.00; *p* = 0.31). Participants with a higher level of education are significantly more stressed (*t*(247) = −2.61; *p* < 0.05), while there are no statistically significant differences in stress by biological sex (*t*(247) = −1.78; *p* = 0.077) or ease of access to green spaces (*t*(247) = 0.40; *p* = 0.68). Women achieve higher psychophysical recovery than men (*t*(247) = 4.10; *p* < 0.001) and participants with lower (non-university) level of education recover more (*t*(247) = 2.04; *p* < 0.05), while there are no statistically significant differences in psychophysical recovery by ease of access to green spaces (*t*(247) = 0.02; *p* = 0.98).

### 3.2. Effects of Initial Stress and Mental Wellbeing on Psychophysical Recovery After Barefooting Activity

Controlling for age, and familiarity with nature, a multivariate logistic regression analysis was conducted to examine the likelihood of high psychophysical recovery after barefooting activity among people with different levels of perceived stress (Model 1). The overall model explained 7.4% (Nagelkerke’s R^2^) of the variance. Perceived stress in the last two weeks was not a significant predictor (Exp (B) = 0.97, 95% IC [0.92, 1.01]), suggesting that different levels of stress in the last two weeks were not associated with an increased likelihood of belonging to the high recovery group after barefooting activity (Table 2). A further multivariate logistic regression analysis was conducted to examine the likelihood of high psychophysical recovery after barefooting activity among people with different levels of mental wellbeing controlling for age and familiarity with nature (Model 2). The overall model explained 17.8% (Nagelkerke’s R^2^) of the variance. Mental wellbeing was a significant predictor (Exp (B) = 1.11, 95% IC [1.06, 1.16]), suggesting that higher levels of this psychological construct were associated with a greater likelihood of significant recovery after barefooting activity (Table 2).

### 3.3. The Moderating Effect of Biological Sex, Education Level and Ease of Access to Green Spaces

Adjusting for age and familiarity with nature, multivariate logistic regression models were conducted to examine whether the interactions between stress and biological sex, education level, and ease of access to green spaces could predict the likelihood of good psychological recovery. In regarding the first model, the interaction between stress and sex was not significant (Exp(B) = 1.04, 95% IC [0.95, 1.15]), indicating that the effect of stress on recovery did not vary significantly by biological sex. Similarly, the interaction between stress and education level was not significant (Exp(B) = 1.05, 95% IC [0.95, 1.16]), indicating that the effect of stress on recovery did not vary significantly by education level. Finally, the interaction between perceived stress and ease of access to green spaces was not significant (Exp(B) = 0.97, 95% IC [0.88, 1.06]), indicating that the effect of stress on psychological recovery did not vary significantly by ease of access to green spaces (Table 3). Adjusting for age and familiarity with nature, multivariate logistic regression models were also conducted to examine whether the interactions between mental wellbeing and biological sex, education level, and ease of access to green spaces could predict the likelihood of significant psychological recovery. In the first model, the interaction between mental wellbeing and sex was not significant (Exp(B) = 1.01, 95% IC [0.93, 1.11]), indicating that the effect of mental wellbeing on recovery did not vary significantly by sex. Also, the interaction between mental wellbeing and education level was not significant (Exp(B) = 0.95, 95% IC [0.86, 1.05]), indicating that the effect of mental wellbeing on recovery did not vary significantly by education level. Finally, the interaction between mental wellbeing and ease of access to green spaces was not significant (Exp(B) = 1.04, 95% IC [0.95, 1.14]), indicating that the effect of mental wellbeing on psychological recovery did not vary significantly by ease of access to green spaces (Table 3).

## 4. Discussion

In this study, we aimed to investigate how individual baseline conditions and contextual factors influence the likelihood of achieving psychophysical recovery through immersive barefoot walking in nature. Specifically, our study addressed three key questions. First, we examined whether baseline stress levels could predict the likelihood of high psychophysical recovery: participants who reported low perceived stress in the two weeks prior to the activity were just as likely to report high levels of recovery as those who felt highly stressed. Second, we explored whether higher baseline mental wellbeing predicted the likelihood of reporting high recovery: participants with higher initial mental wellbeing was more likely to report stronger psychophysical recovery than those with lower mental wellbeing. Third, we examined whether education level (as proxy for SES), biological sex, and ease of access to natural green spaces moderated the relationship between baseline stress levels and baseline mental wellbeing, respectively, and psychophysical recovery, and found no significant moderating effects.

### 4.1. Baseline Stress Level Predicts Psychophysiological Restoration

Contrary to the original assumptions, the perceived stress level in the previous two weeks was not a significant predictor of psychophysical recovery after barefooting. This result suggests that, in this study, the perception of short-term stress is not a decisive factor for the immediate benefits of barefooting. The classic studies by Ulrich and colleagues [9] have fundamentally contributed to our understanding of the restorative power of nature, showing that spending time in a natural environment promotes more effective recovery from stressful conditions compared to an urban environment. However, it should be noted that their methodology involved an experimental manipulation of stress that was artificially and uniformly elevated in participants. Our study takes a different approach: instead of experimentally altering stress levels, we measured the subjective perception of daily stress as naturally experienced by participants over the previous two weeks. This allows us to study the effects of barefooting in conditions closer to real life, but at the same time, it could explain why there was no significant effect in terms of perceived stress levels. Similarly, the contributions of Kaplan [8] have provided a valuable theoretical framework for understanding how contact with nature promotes recovery from states of ‘mental fatigue’, a predominantly cognitive construct associated with the depletion of attentional resources. The stress measure used in our work assesses the perception of unpredictability, uncontrollability and overload in one’s life, aspects that relate to a broader experience than just the cognitive dimension. In this sense, our study broadens the perspective and focuses not so much on the recovery of attentional resources as on the impact of perceived stress as a general condition of daily life. In line with our findings, the study by Beute and De Kort [37] found no significant effects of the natural environment on immediate stress reduction. However, the authors point out that these results may depend on the type of design used, which focuses exclusively on the immediate effects of the environment. Indeed, it is possible that the immediate benefits of contact with nature, when repeated and consolidated over time, translate into a change in stress levels. In light of our findings, it may be interesting for future studies to further investigate the distinction between acute and chronic stress. As Olafsdottir and colleagues [38] have shown, a 40 min walk in nature was able to attenuate chronic stress responses, while there were no significant differences in acute stress responses. This result underlines that the benefits of contact with nature are mainly seen in persistent and chronic stress processes and not in response to acute or short-term stimuli.

### 4.2. Baseline Wellbeing Level Predicts Psychophysiological Restoration

Our findings show that higher baseline levels of mental wellbeing significantly predict greater psychophysical recovery following barefoot activity in a non-clinical population. This extends existing literature by highlighting the role of nature not only in alleviating psychological deficits but also in sustaining and enhancing wellbeing in individuals who are already mentally healthy [39,40,41]. Previous research has primarily focused on the therapeutic effects of nature exposure among individuals with common mental disorders, e.g., [14,15], where significant improvements are often observed relative to an impaired baseline. In contrast, our results indicate that heightened wellbeing at baseline confers an advantage, amplifying the absolute restorative benefits derived from immersive activities such as barefoot walking. This interpretation aligns with prior evidence showing that the restorative impact of natural environments depends on one’s initial psychological state. Barton and Pretty [42] found that green exercise interventions have stronger effects on both physical and psychological outcomes among individuals with higher baseline wellbeing. Similarly, Tyrväinen et al. [43] observed that exposure to urban green environments produced greater benefits for individuals who already reported higher levels of happiness and psychological stability. Together, these findings suggest that a favorable baseline condition facilitates deeper engagement with nature and maximizes its restorative potential. At the same time, it is important to acknowledge that individuals with common mental disorders, although benefiting symptomatically from nature exposure, often report reduced enjoyment and increased discomfort in natural environments [14]. Such barriers may constrain the depth of engagement and limit the psychophysical gains of the experience. In contrast, participants in our study with high baseline wellbeing likely experienced fewer psychological obstacles, enabling a fuller, more embodied interaction with the natural setting. This absence of psychological friction may account for the greater recovery outcomes observed, underscoring the importance of baseline wellbeing as a moderator of nature’s restorative effects. Barefoot activity represents a unique, intense and intimate engagement with nature. Unlike general outdoor activities (e.g., walking in the park or picnicking), barefoot walking involves direct tactile stimulation, physical grounding, and solitary presence, qualities that can promote deeper physiological relaxation and an enhanced sense of connectedness with the environment, as theorized in biophilia [44]. For people who are already well, this type of multisensory, contemplative exposure may serve not only as a restorative experience, but also as a mechanism for maintaining and optimizing wellbeing [29,45].

### 4.3. Moderator Role of SES Level, Biological Sex, and Accessibility to Natural Green Spaces

Our analysis examined whether the associations between stress, mental wellbeing, and psychological recovery after barefoot activity were moderated by sex, education level (used here as a proxy for socioeconomic status), and ease of access to green space. None of the tested interactions reached statistical significance, suggesting that the observed effects, particularly the contribution of high baseline mental wellbeing to restorative outcomes, may be relatively stable across sociodemographic subgroups. The absence of a moderating effect of sex is consistent with findings in the broader nature–health literature, which indicate that men and women often derive comparable psychological benefits from direct engagement with natural environments [46,47]. While some studies have reported sex differences, often showing stronger stress-reduction effects among women [48], these variations appear to be influenced by contextual factors such as perceived safety, cultural roles, or the type of activity undertaken. In the present study, barefoot walking represented a direct and embodied interaction with nature, which may have reduced the likelihood of gendered patterns in the restorative response. Education level, as a proxy for socioeconomic status (SES), was also not found to moderate the relationship between mental wellbeing and psychological recovery. Education is widely used as an SES indicator in public health and epidemiological research [34], and disparities in SES are often linked to inequalities in access to health-promoting resources, including nature [49]. The lack of moderation observed here could suggest that, when individuals are given the opportunity to engage in a structured and immersive experience, psychological recovery is not strongly differentiated by SES. This interpretation aligns with environmental health literature suggesting that inclusive, low-cost nature-based practices may help reduce health inequalities [50,51]. However, it is important to emphasize that our results relate to a sample that independently chose to take part in a barefooting experience and is therefore self-selected and difficult to generalize to the total population. It may exclusively reflect regular visitors to parks and nature activities. Finally, ease of access to green space did not moderate the relationship between stress, mental wellbeing, and recovery. Prior studies have shown that residential proximity to green space is generally associated with lower stress, better mood, and improved mental health outcomes [7,52]. Yet, proximity alone does not guarantee use, nor does it determine the quality of the experience. Recent frameworks emphasize that the depth, quality, and intentionality of contact with natural environments may play a stronger role than simple accessibility [53]. Our results align with this perspective, suggesting that even individuals with limited everyday access to green spaces may benefit from a meaningful and multisensory interaction with nature, provided they begin with high levels of mental wellbeing.

### 4.4. Limitations and Future Directions

This study has several limitations that should be considered. First, its cross-sectional design prevents clear causal inferences about the relationship between barefoot walking and restorative outcomes. Second, the absence of a control condition limits the ability to determine whether barefoot walking provides specific benefits beyond those associated with other forms of nature contact. Third, the sample was self-selected from individuals who voluntarily enrolled in the barefoot trail, often paying a registration fee, which may indicate a pre-existing motivation or positive attitude toward the activity and introduce potential self-selection and expectancy biases. Fourth, the study relied exclusively on self-report measures, which are susceptible to subjective interpretation and social desirability bias; future research would benefit from including physiological or behavioral indicators of recovery (e.g., cortisol levels, heart rate variability). Fifth, the activity was experienced only once and in a relatively short time frame, making it difficult to evaluate whether the observed effects would persist or change with repeated or longer-term engagement. Finally, the study was conducted in a specific geographic and cultural context, which may restrict the external validity of the findings.

Future research should use longitudinal and experimental designs, include appropriate control groups, and recruit more diverse samples, including clinical populations with lower baseline wellbeing, to clarify the conditions under which barefoot walking may provide restorative benefits. Integrating objective physiological measures would also strengthen conclusions about psychophysical recovery and reduce reliance on self-report.

## 5. Conclusions

This study adds to the growing body of research on the restorative effects of nature by examining how baseline individual conditions and contextual factors influence recovery outcomes from barefoot activity. Our results show that people with higher initial levels of mental wellbeing, regardless of sociodemographic background or environmental accessibility, are more likely to experience the maximum recovery benefits that barefooting in nature can provide. In contrast, recent stress was not a significant predictor of recovery, suggesting that, for barefooting, mental wellbeing rather than short-term stress best predicts psychophysical recovery. These findings expand the literature by indicating that immersive, multisensory practices such as barefoot walking may serve not only as therapeutic strategies for those in distress but also as ways to sustain and optimize wellbeing in already healthy populations. The lack of moderating effects from sex, education, and access to green space further suggests that the restorative potential of barefoot walking may be broadly accessible across different sociodemographic groups, at least among individuals who voluntarily participate in such activities. Practically, these results highlight the potential of barefoot walking trails and similar immersive practices as inclusive, low-cost interventions that can be implemented in public green spaces to promote mental health and wellbeing. However, given the cross-sectional design, reliance on self-report measures, and the unique and self-selected nature of the experience, our conclusions remain tentative. Future research using longitudinal and experimental designs, incorporating physiological indicators of stress, and including more diverse populations will be essential to clarify the mechanisms through which barefoot walking exerts its effects and to establish its role within broader strategies for health promotion and environmental design.

## Figures and Tables

**Table 1:** Descriptive statistics for the total sample.

Variables	Sample	Mean (sd)	Skewness	Kurtosis	*t*-Score
Wellbeing	Total (*n* = 249)	43.79 (6.50)	−0.28	0.19	
	Female (*n* = 144)	44.15 (6.14)			0.99
	Male (*n* = 105)	43.30 (6.97)			
	Low education level (*n* = 102)	44.21 (6.81)			0.83
	High education level (*n* = 147)	43.50 (6.29)			
	Access to green space (*n* = 129)	44.19 (6.44)			1.00
	No access to green space (*n* = 120)	43.36 (6.57)			
Psychophysical recovery	Total (*n* = 249)	33.72 (6.49)	−1.15	1.95	
	Female (*n* = 144)	35.13 (6.11)			4.10 **
	Male (*n* = 105)	31.79 (6.53)			
	Low education level (*n* = 102)	34.75 (6.90)			2.04 *
	High education level (*n* = 147)	33.01 (6.11)			
	Access to green space (*n* = 129)	33.73 (6.71)			0.02
	No access to green space (*n* = 120)	33.71 (6.27)			
Stress	Total (*n* = 249)	17.77 (5.80)	−0.26	−0.22	
	Female (*n* = 144)	17.21 (5.81)			−1.78
	Male (*n* = 105)	18.53 (7.72)			
	Low education level (*n* = 102)	16.59 (6.29)			−2.61 *
	High education level (*n* = 147)	18.58 (5.30)			
	Access to green space (*n* = 129)	17.91 (6.18)			0.40
	No access to green space (*n* = 120)	17.61 (5.37)			

Note: * *p* < 0.05; ** *p* < 0.001.

**Table 2:** Principal effects.

Model	B	S.E.	Wald	gl	*p*	Exp(B)
**Model 1**						
Stress	−0.03	0.02	1.70	1	0.192	0.97
**Model 2**						
Wellbeing	0.10	0.02	19.97	1	0.000 **	1.11

Note: ** *p* < 0.001.

**Table 3:** Interaction effects.

Model	B	S.E.	Wald	gl	*p*	Exp(B)
**Model 1**						
Stress × Sex	0.04	0.05	0.74	1	0.390	1.04
Stress × Education level	0.05	0.05	1.02	1	0.312	1.05
Stress × Accessibility to green spaces	−0.03	0.05	0.54	1	0.463	0.965
**Model 2**						
Wellbeing × Sex	0.01	0.05	0.06	1	0.770	1.01
Wellbeing × Education level	−0.05	0.05	1.14	1	0.284	0.948
Wellbeing × Accessibility to green spaces	0.04	0.05	0.72	1	0.395	1.04

## Data Availability

The data presented in this study are available on request from the corresponding author due to being part of the ALCOTRA NAT.SENS Project 2014–2020.
